# Dynamic and non-contact 3D sample rotation for microscopy

**DOI:** 10.1038/s41467-018-07504-3

**Published:** 2018-11-28

**Authors:** Frederic Berndt, Gopi Shah, Rory M. Power, Jan Brugués, Jan Huisken

**Affiliations:** 1 Max Planck Institute of Molecular Cell Biology and Genetics, Pfotenhauerstr. 108, 01307 Dresden, Germany; 2 Max Planck Institute for the Physics of Complex Systems, Nöthnitzer Str. 38, 01187 Dresden, Germany; 3 Center for Systems Biology Dresden, Pfotenhauerstr. 108, 01307 Dresden, Germany; 4 European Molecular Biology Laboratory, Carrer del Dr. Aiguader, 88, 08003 Barcelona, Spain; 5 Morgridge Institute for Research, 330 N Orchard St, Madison, WI 53715 USA

## Abstract

Precise sample orientation is crucial for microscopy but is often performed with macroscopic tools and low accuracy. In vivo imaging of growing and developing samples even requires dynamic adaptation of the sample orientation to continuously achieve optimal imaging. Here, we present a method for freely positioning a sample in 3D by introducing magnetic beads and applying a magnetic field. We demonstrate magnetic orientation of fixed mouse embryos and *artemia*, and live zebrafish embryos and larvae on an epi-fluorescence microscope and on a light-sheet system for optimal imaging.

## Abstract (web summary)

Sample orientation is crucial to ensure optimal image quality in light microscopy. Here the authors enable multi-axis orientation of fixed mouse embryos and shrimp, and live zebrafish embryos and larvae by introducing magnetic beads and rotating the sample with a magnetic field in a microscope.

## Introduction

Imaging biological samples with light microscopy often requires a certain sample orientation for obtaining optimal image quality. Absorbing and scattering tissues such as pigments, eyes or yolk can obscure the area of interest in certain orientations. Since the sample is embedded prior to imaging, its orientation is typically fixed for the duration of the experiment and can no longer be adjusted when mounted in the microscope. During in vivo experiments the optical properties of the sample and the ideal imaging angle may even change. Hence, to study living organisms under optically optimal conditions, a technique to dynamically adjust sample orientation in the microscope is needed.

**Fig. 1:**
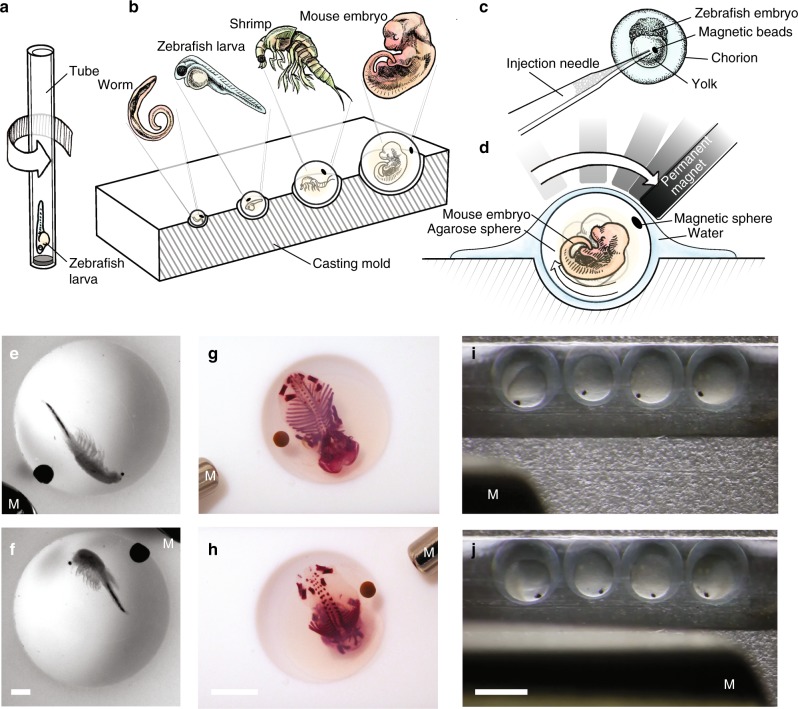
Embedding strategies for various samples and 3D non-contact orientation. **a** Schematic of the uniaxial rotation of a fish larva in a SPIM system. **b** Drawing showing the embedding of different organisms in differently sized agarose spheres by using a casting mold with hemispherical wells. **c** Drawing showing the injection of magnetic beads into the yolk of the zebrafish embryo. **d** Drawing showing a mouse embryo embedded in an agarose sphere rotated by a permanent magnet. **e**, **f** Bright-field images of a fixed artemia spec. and **g**, **h** a fixed, skeletal stained mouse embryo (E15.5) embedded in an agarose sphere and rotated by a permanent magnet (M). Scale bar, 1 mm and 5 mm, respectively. **i**, **j** Bright-field images of injected zebrafish embryos rotated by a permanent magnet. Scale bar, 1 mm

Sample orientation techniques have been developed for high-throughput applications using microfluidic systems^1^ and for analysis of expression patterns in zebrafish larval brains by manually inverting the sample holder^2^. However, these methods still lack adaptive reorientation of the sample in the microscope during the experiment. Optical methods have been used to position and orient single cells and small worm embryos (*Pomatoceros lamarckii*, 60 µm) for light microscopy^3–5^. However, forces exerted by optical methods are not sufficient to position larger model organisms commonly used in developmental biology, for which sample orientation is even more important, and higher light doses would damage the sample. In some implementations of light-sheet microscopy (or Selective Plane Illumination Microscopy (SPIM)^6^) a single degree of adaptive orientation of the sample is offered by a vertical rotational axis (Fig. 1a). However, this uniaxial rotation is insufficient for total control over three-dimensional orientation. Here, we present a non-contact method to adaptively orient fixed and live specimens in a microscope. We show that after the insertion of magnetic beads, the sample can be freely positioned in 3D during the experiment by applying a magnetic field. We demonstrate this technique in zebrafish embryos and larvae, *artemia* and fixed mouse embryos.

## Results

### Embedding and injection of magnetic beads

To rotate a sample with magnetic forces, a magnetic handle needs to be inserted into or attached to the sample. We created small magnetic agarose spheres (Supplementary Figure 1 and Supplementary Method 1), which can be embedded together with the sample in an enclosing agarose sphere. To accommodate a variety of sample sizes (500 µm–12 mm), we created hemispherical molds and spherical injection molds with different diameters (Fig. 1b and Supplementary Figure 2). The spherical shape of the embedded sample made later rotation of the sample into the preferred orientation easy.

Alternatively, embryos of some species are encapsulated in a fluid filled chorion and develop on top of the yolk, making it easy to rotate them inside the chorion. In the case of the zebrafish embryo, superparamagnetic beads could be directly injected into the yolk with a microinjection device (Fig. 1c and Supplementary Method 2). We monitored injected embryos with and without magnetic field for 4 days and found no visible delay or defect in development when compared to non-injected wildtype larvae (Supplementary Figure 3). As the fish developed, the beads stayed in the remaining yolk, close to the yolk extension, still permitting magnetic orientation of the larva.

Embedding various specimens in agarose spheres and rotating them was straightforward (Fig. 1d). We oriented 6 mm-sized *artemia* (Fig. 1e, f) and 12 mm-sized mouse embryos (Fig. 1g, h, Supplementary Movies 1, 2) in a non-contact manner by moving a permanent magnet over the sample sitting in one of the molds. It became apparent that especially for such large samples, the ability to freely rotate the sample is instrumental when multiple areas need to be imaged that cannot be reached in one fixed orientation. Zebrafish embryos rotated smoothly within their chorion upon application of a magnetic field (Fig. 1i, j, Supplementary Movie 3). The rotation results from the attraction of the magnetic beads by the permanent magnet. The force applied led to a translation and a rotation of the sample, thus minimizing the distance between the beads and the magnet.

### Microscope insert for magnetic rotation of zebrafish larva

For dynamic control of the magnetic field we then used electromagnets (Supplementary Figure 4). As the force acting on a superparamagnetic bead is proportional to the magnetic field gradient^7^, the core of the electromagnets were sharpened to create a sufficiently strong magnetic field gradient even with a moderate current (300 mA), keeping the heating of electromagnets to a minimum. With two such electromagnets a sample can be easily rotated around one axis, e.g. in a tube or a capillary.

**Fig. 2:**
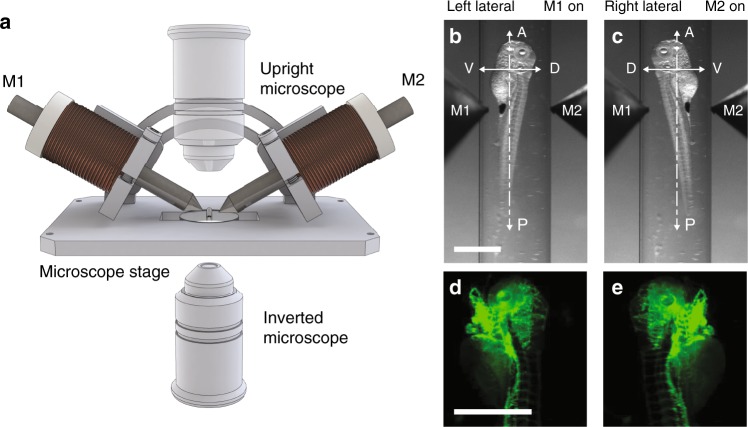
Insert for multi-view imaging on a single-view microscope. **a** Schematic showing the insert holding two electromagnets (M1 and M2) on a microscope stage. **b**–**e** Bright-field (**b**, **c**) and fluorescence (**d**, **e**) images of a 5dpf Tg(kdrl:GFP) zebrafish larva rotated about its anterior-posterior axis by providing power to electromagnet M1 and M2, respectively. Scale bar, 1 mm

We developed an insert consisting of a plate and an arc holding two electromagnets that can be easily adapted to any upright or inverted light microscope (Fig. 2a). Two-view imaging of a zebrafish larva (5dpf, *Tg(kdrl:GFP)*^8^) on an upright epi-fluorescence microscope is shown. The magnet orientation can be adapted continuously by sliding the magnets along the arc. The rotation of the zebrafish larva worked best with an angle of about 35° between the electromagnet and the plate.

The zebrafish larva was embedded in a glass capillary, positioned at the intersection of the magnet axes and oriented about its anterior-posterior axis. When switching on one magnet, the larva was translated towards the wall of the glass capillary and rotated about 180° towards the magnet (Supplementary Movie 4). When the magnet was switched off, the larva was released from the force and settled in its resting position (Fig. 2b–e). A 180° rotation from the left lateral to the right lateral resting position took no longer than 10 s with 1 A current, illustrating that our system can add multi-view capabilities to any conventional microscope.

### Tetrahedral electromagnet assembly for 3D sample orientation

**Fig. 3:**
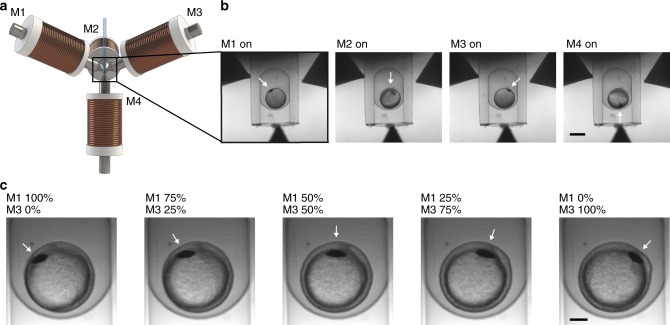
Tetrahedral electromagnet geometry for 3D orientation of injected zebrafish embryos. **a** Four electromagnets (M1, M2, M3, M4) in a tetrahedral geometry were assembled around the sample tube. **b** Bright-field images of a zebrafish embryo encapsulated in the sample tube. The embryo was oriented by applying a magnetic field by magnet M1, M2, M3, and M4. Scale bar, 500 μm. **c** The zebrafish embryo was rotated continuously from magnet M1 to magnet M3 by changing the ratio of the applied currents between the two magnets. Scale bar, 200 μm

To orient the sample in three dimensions, we used four electro-magnets arranged in a tetrahedral geometry with the sample in the center (Fig. 3a). We inserted the magnets such that they neither collide with the objectives nor interfere with imaging. A zebrafish embryo injected with magnetic beads was embedded in an FEP tube. The inner diameter (1.0 mm) was slightly smaller than the chorion (~1.2 mm) to hold the zebrafish in the FEP tube (Fig. 3b). By sequentially switching between four magnets, the embryo was rotated in a non-contact manner and positioned in four different orientations given by the tetrahedral arrangement of electromagnets (Fig. 3b, Supplementary Movie 5). We measured the performance of the system and found that a rotation of the zebrafish embryo from one magnet to another one (109.5°) took <30 s at a current as low as 300 mA. This transition time was inversely proportional to the applied current and could therefore be tuned according to the application (Supplementary Figure 5). A low current led to slow and homogenous rotation whereas a high current led to a rapid reorientation. After releasing the embryo from the magnetic force the zebrafish retracted for <10 s and settled at its final position. We found that the embryo remained stable in its settled position for over a minute, longer than the time it needs to take a three-dimensional stack of the whole embryo on a SPIM system (Supplementary Figure 5).

Intermediate positions can be accessed by applying currents to two or more magnets simultaneously. The embryo orients along the resulting magnetic field. Hence, by changing the ratio of the currents applied to the two magnets, the embryo can be rotated continuously either to align with the corresponding orientations above or at any intermediate position (Fig. 3c).

### Rotation of zebrafish embryos in a multi-axis SPIM setup

**Fig. 4:**
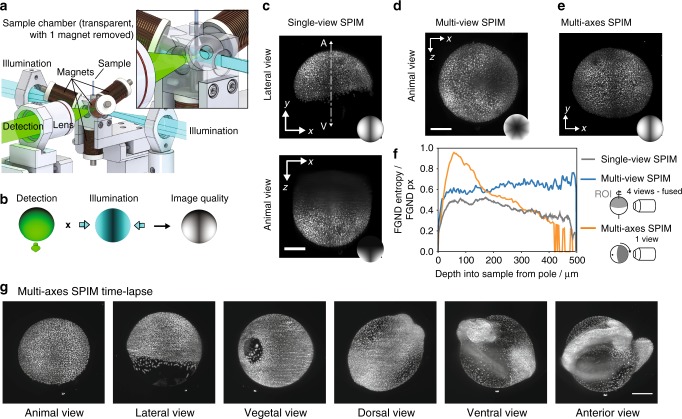
Non-contact 3D orientation technique adapted to a SPIM system. **a** Schematic of the SPIM setup with the tetrahedral electromagnets arranged around the sample tube. The magnets were held by the sample chamber and the sample was illuminated by a light sheet through two windows. Fluorescence was detected through a third window by a detection objective. The sample chamber and the detection objective were motorized to move the sample through the light sheet and to correct for the different path lengths in air and water. **b** Schematic representation of the obtained image quality in SPIM imaging, which is influenced by the sample orientation relative to the detection and illumination objectives. **c** Maximum intensity projection of a single-view stack from a histone labeled zebrafish embryo. Scale bar, 200 μm. Inset: schematic representation of the image quality obtained by a single view. **d** Maximum intensity projection obtained by multi-view SPIM imaging of four views (0°, 45°, 180°, and 225°). The fusion of the four views shows a homogenous resolution along the equator but a decreasing resolution towards the cap (rotational axis). Scale bar, 200 μm. Inset: schematic representation of the image quality obtained by multi-view fusion of four views. **e** Maximum intensity projection of a single-view stack obtained with the multi-axis SPIM technique. The view on the animal pole shows the improved image quality of the animal pole (ROI) obtained by orienting the animal pole towards the detection objective by the multi-axis sample orientation technique. Scale bar, 200 μm. Inset: schematic representation of the image quality obtained with the multi-axis SPIM technique. **f** Local entropy as a measure of the image quality of the single-view, multi-view, and multi-axis data along the animal-vegetal axis. **g** Stills from a time-lapse of a developing, histone labeled zebrafish taken in different orientations to watch key events in the respective optimal orientation (Supplementary Movie 6). Scale bar, 200 μm

To image a developing zebrafish in its optimal orientation with high resolution and low photo-toxicity, we implemented the tetrahedral electromagnet configuration around the sample chamber in a SPIM setup (Fig. 4a, additional SPIM geometries for high-NA imaging can be found in Supplementary Figure 6). Typically, SPIM provides only a single axis of rotation for multi-view imaging^6,9^ (Fig. 1a) and we considered whether the additional degrees of freedom in our setup could lead to improved coverage of the sample. It is worth noting that SPIM requires special attention to the sample orientations as the perpendicular illumination and detection axes both need obstruction-free access to the area of interest.

In SPIM, the view directly facing the detection objective can be captured well, but the image quality suffers from obliquely incident illumination in spherical or ellipsoidal samples (e.g. zebrafish, drosophila embryos). At the same time, the orthogonal views facing the illumination objectives are well illuminated but poorly resolved due to the longer optical path through tissue for detection. Therefore, the best image quality is achieved in the region between the illumination and detection objectives, which can be well illuminated and fluorescence detected with minimal aberrations^10^ (Fig. 4b). When rotating the sample in a conventional, single rotational-axis system, uniform coverage is achieved only along the equator. The image quality at the poles is always poor owing to its inaccessibility for illumination and detection.

During early development, the embryo is in an orientation with the animal pole on top of the yolk (north pole). The animal pole is therefore only poorly accessible for the conventional SPIM since the rotational axis of the SPIM is parallel to the animal-vegetal axis (A-V-axis) (Fig. 4c). To reconstruct the animal pole, we imaged an injected zebrafish embryo (*Tg(h2afva:h2afva-GFP)*^11^) in four different orientations by rotating the sample tube, registering and fusing these non-ideal views. (Fig. 4d). In contrast, in our multi-axis SPIM mode we were able to orient the animal pole facing the detection objective irrespective of its initial orientation and a single stack was sufficient to image the entire region of interest (ROI) (Fig. 4e).

We took the image stack captured after rotation and the result of the multi-view fusion and calculated the local entropy of each plane in the stack as a measure of image quality. The multi-axis rotation delivers superior image quality at the poles of the embryo, regions that are never accessible in the conventional single-axis rotation (Fig. 4f, Supplementary Figure 7). The superior optical coverage obtained in mesoscopic samples via multi-view fusion is well characterized^10^. As one goes deeper into the stack we observe an increase in image quality in the fused images consistent with this improvement as the combined views provide superior coverage away from the pole. Thus, by positioning the sample in its ideal orientation we achieve superior image quality compared to the multi-view SPIM, with less views needed, reducing light exposure and acquisition time.

To illustrate dynamic sample rotation during sample development, we performed time-lapse imaging of an injected, developing zebrafish embryo (*Tg(H2A-GFP)*^11^). To always have the optimal sample orientation for observing the key events during development, we oriented the zebrafish by magnetic forces and rotation of the tube. We captured, e.g. the cellular dynamics from the animal view, collective epiboly movement from the lateral side, the dorsal convergence, and extension movements from dorsal, and head and tail formation from anterior (Fig. 4g, Supplementary Movie 6). During early stages (0–10hpf, corresponding to 1-cell stage to 90% epiboly), the embryo had a nearly spherical shape, making rotation easy. At later stages, with the emergence of pronounced head and tail structures and the convergence of the dorsal side, the sample geometry limited rotations about the anterior-posterior and the dorsal–ventral axis. We adapted the orientation about the left-right axis at these stages by rotating the tube. With both techniques combined, we could adapt the orientation of a single zebrafish embryo in the microscope to watch key events that could otherwise only be captured in several separate experiments.

## Discussion

We have developed a non-contact method to orient specimens in a microscope by introducing a magnetic handle and applying an external magnetic field. Our technique relies on magnetic forces applied on beads injected into an organism or attached to it. It is important to note that high magnetic forces can lead to the deformation of the sample or a translation of the beads within the yolk, which could potentially damage the sample. We averted such damage by using minimal forces over a short time. The magnetic forces can lead also to a translation of the sample within the chorion for zebrafish embryos or the embedded sample within the mold. However, additional translation is necessary to center the region of interest after rotation anyway. Since the electromagnet tips are thinned to provide maximum field gradients, the additional steric hindrance is negligible when considering the solid angle around the samples needed for optical access (i.e. objective lens).

High-throughput methods^12^, which currently do not offer any rotational axis for sample orientation, will greatly benefit from our non-contact sample orientation technique. All samples can be similarly oriented, increasing the screening sensitivity and specificity. Likewise, photo-manipulation techniques particularly benefit from the full flexibility to orient the sample such that the intervention can be targeted precisely to the desired region.

## Methods

### SPIM setup

The SPIM setup consisted of an Olympus XLFLUOR (4 × /0.28) lens for detection and two lenses (AC254-060-A, Thorlabs, USA) aligned orthogonal to the detection for double-sided light-sheet illumination and used to relay the light sheets (488 nm, Coherent Sapphire laser 488-30 CDRH) generated by a pair of cylindrical lenses (ACY254-050-A, Thorlabs, USA). The sample was illuminated alternately from two sides controlled via a flipper mirror (8892-K-M, New Focus, USA). The sample chamber was 3D-printed and had two windows for illumination and one window for detection of the fluorescence. The electromagnets were held by the sample chamber and oriented in a tetrahedral geometry. The magnets were inserted such that they neither collide with the objectives nor interfere with imaging. The applied current was remotely controlled via a programmable power supply (QL355P, Aim-TTi, United Kingdom) and a manual switch. The focus of the four electromagnet-tips coincided with the sample. The sample was inserted from the top within an FEP tube orthogonal to the detection objective and the illumination lenses. For sample scanning, the sample chamber was mounted on a motorized linear stage (M111.1DG, Physik Instrumente, Germany) and moved relative to the detection objective. To correct for the different path length in water and air, the detection objective was also placed on a linear stage (M111.1DG, Physik Instrumente, Germany) and moved accordingly. For bright-field illumination an LED (CCS TH-27/27-SW, Stemmer imaging GmbH, Germany) was placed behind the sample. The bright-field and fluorescence signals were detected with an sCMOS camera (Zyla 5.5, Andor, United Kingdom).

### Electromagnets

The electromagnets were custom-built and consisted of a magnetic core made from HyMu 80 alloy (National Electronic Alloys, USA) and a solenoid (Supplementary Figure 4). The bobbin was made from Teflon and a high resistance wire was wound around it hundreds of times to create a strong magnetic field. The magnetic core had a diameter of 6 mm and was tapered to create a strong magnetic field gradient. The inner diameter of the bobbin was 6 mm into which the magnetic core could be inserted.

### Injections of zebrafish embryos

See Supplementary Methods 2 ‘Washing and injection of superparamagnetic beads’. In brief, superparamagnetic beads (Dynabeads MyOne Carboxylic Acid, Invitrogen, USA) with a diameter of 2.8 µm were injected with standard glass needles into the yolk of the zebrafish embryo. To avoid dispersion of beads, injections were performed at very low pressure (5–10 psi) and long injection duration (100–150 ms). A volume of 1 nl of a 5 mg per ml bead solution was injected for rotation of the embryo.

### Insert for a stereoscope and inverted microscopes

The magnetic manipulator insert was built from an aluminum plate with a size of 92.5 × 64 mm fitting on standard microscope stages. On this plate, an arc was mounted, holding two electromagnets that can be freely slid along the arc to control the angle between the magnets and the sample. The sample was either embedded in a glass capillary for mounting on an upright microscope or placed on a coverslip and held by a Teflon holder to study the sample on an inverted microscope.

### Zebrafish samples

Zebrafish (*Danio rerio*) adults and embryos were kept at 28.5 °C and were handled according to established protocols^13^. Transgenic lines *Tg(kdrl:GFP)*^8^ and *Tg(h2afva:h2afva-GFP)*^11^ were used. For SPIM imaging, the zebrafish embryos were either embedded in small (inner diameter 1.0 mm, outer diameter 1.6 mm) FEP tubes in E3 or in larger (inner diameter 1.6 mm, outer diameter 2.4 mm) FEP tubes in 1.0% low-melting-point agarose (Sigma).

For imaging the zebrafish larvae on the stereoscope, the embryos were treated with 0.2 mM 1-phenyl 2-thiourea (Sigma) at 24 h post fertilization to inhibit melanogenesis. During imaging, the samples were anaesthetized with 200 mg per l Tricaine (Sigma) and embedded inside glass capillaries in E3 containing 200 mg per l Tricaine. All animals were treated in accordance with EU directive 2011/63/EU as well as the German Animal Welfare Act.

### Mouse embryo samples

Wildtype mouse embryos were dissected out of the mother’s uterus on embryonic day E15.5 and transferred in PBS. They were immediately fixed in 95% ethanol and stained with Alizarin red. The embryos were cleared afterwards in KOH overnight and transferred to a glycerol ethanol solution (1:1) for storage, following the protocol by D. Rigueur and K.M. Lyons^14^.

For imaging, the embryos were placed in PBS to remove the glycerol ethanol solution and embedded in a 1.5% low-melting-point agarose (Sigma) sphere along with a magnetic agarose sphere using our spherical injection mold.

### Shrimp samples

*Artemia* spec. were fixed in PFA at 4°C for 12 h. For imaging, each embryo was embedded in a 1.5% low-melting-point agarose (Sigma) sphere along with a magnetic agarose sphere using our custom-designed hemispherical mold.

### Sample handling for data acquisition on the multi-axes SPIM

We embedded the zebrafish embryo (*Tg(h2afva:h2afva-GFP)* or *Tg(H2A-GFP)*^11^) in an FEP tube (inner diameter 1.6 mm, outer diameter 2.4 mm) with 1.0% low-melting-point agarose (Sigma). For recording the multi-view SPIM data we manually rotated the sample tube. We took two views 45° apart from both sides of the sample^10^. The four different angles (0°, 45°, 180°, and 225°) were registered and fused with the feature-based registration using the nuclei as features^15,16^.

For recording the multi-axis stack we oriented the same injected embryo within its chorion towards the detection objective with the four electromagnets. Before recording a stack, we switched the magnet off to avoid any deformations by the applied force. Switching off resulted in a slight reorientation in a new settle position. After a few seconds, when the embryo had settled in its new resting position, a 3D-stack of the embryo was acquired. To capture the time-lapse data, we oriented the injected embryo by applying magnetic forces and by rotating the sample tube to watch key events during development from the respective optimal orientation.

### Image quality analysis

Image quality is determined on a plane by plane basis as follows. The maximum projection of the image stack is calculated and a background intensity is determined by taking 100×100 pixel sub-images at the four image corners and determining the mean and standard deviation for each individually. These sub-images do not intersect with the embryo throughout the entire stack. To determine the background intensity threshold to apply, we choose the sub-image for which the sum of the mean intensity and three times the standard deviation is largest to account for slight changes in the background across the image. Assuming noise is Gaussian distributed, >99.7% of true background pixels should have intensities below this threshold. This threshold is then applied to each image to produce a foreground mask. The local entropy of each image is subsequently calculated using a 5-pixel radius disk to define the local environment. The entropy of each pixel is a measure of its information content relative to the local environment, thus sharp features with high contrast provide a higher entropy than blurred regions. The radius was empirically chosen to approximately match the size of a single cell and constrain the areas of high entropy to the cell containing regions of the fish as much as possible. To remove any remaining contribution from the background, the resulting entropy image is masked using the foreground mask. To calculate the image quality, the masked entropy is summed for the entire image and normalized by the number of foreground pixels. This ensures that images with low information content do not artificially deliver low image quality. To determine the boundary of the fish defining the zero depth position, we apply the Otsu threshold method to the maximum projection. This determines a lowest intensity that we can, with confidence, state arises from the overlap of the light sheet center (along the detection axis, *z*) with the embryo. The first image in each stack for which any pixel intensity is above this threshold is determined to be *z* = 0. It is worth noting that the embryo becomes visible prior to this image but that this arises from the lower intensity Gaussian tails inefficiently illuminating the embryo. Other planes containing no pixels higher than this threshold are omitted from the entropy analysis, notably the multi-axis data at large depths.

The code is available from the corresponding author upon reasonable request.

## Electronic supplementary material

Description of Additional Supplementary Files

Supplementary Information

Supplementary Movie 1

Supplementary Movie 2

Supplementary Movie 3

Supplementary Movie 4

Supplementary Movie 5

Supplementary Movie 6

## Data Availability

The data that support the findings of this study are available from the corresponding author upon reasonable request. The CAD files of our setups have been uploaded to FigShare (10.6084/m9.figshare.7240457.v1).
