# Supplementary material for: Dynamic and non-contact 3D sample rotation for microscopy
Source: Nat Commun. 2018 Nov 28;9:5025. doi: 10.1038/s41467-018-07504-3 (PMC6261998; doi:10.1038/s41467-018-07504-3)
Supplement: Supplementary file 1 — Description of Additional Supplementary Files [file 41467_2018_7504_MOESM1_ESM.docx]

Description of Additional Supplementary Files

**Supplementary Movie 1:** Fixed and skeletal stained mouse embryo (E15.5) embedded in agarose sphere flipped about posterior anterior axis by permanent magnet.

**Supplementary Movie 2:** Fixed and skeletal stained mouse embryo (E15.5) embedded in agarose sphere rotated about dorsal ventral axis by permanent magnet.

**Supplementary Movie 3:** 4hpf zebrafish embryo oriented by permanent magnet.

**Supplementary Movie 4:** 5dpf zebrafish larva in glass capillary oriented by electromagnets on epifluorescence microscope.

**Supplementary Movie 5:** 5hpf zebrafish embryo embedded in FEP tube and oriented by electromagnets in SPIM setup.

**Supplementary Movie 6:** Time-lapse of an injected and developing zebrafish embryo (Tg(H2A-GFP)) oriented to watch key events during development from the respective optimal orientation
