# Supplementary material for: Dynamic and non-contact 3D sample rotation for microscopy
Source: Nat Commun. 2018 Nov 28;9:5025. doi: 10.1038/s41467-018-07504-3 (PMC6261998; doi:10.1038/s41467-018-07504-3)
Supplement: Supplementary file 2 — Supplementary Information [file 41467_2018_7504_MOESM2_ESM.pdf]

Supplementary Information

Dynamic and non-contact 3D sample rotation for microscopy

Berndt et al.

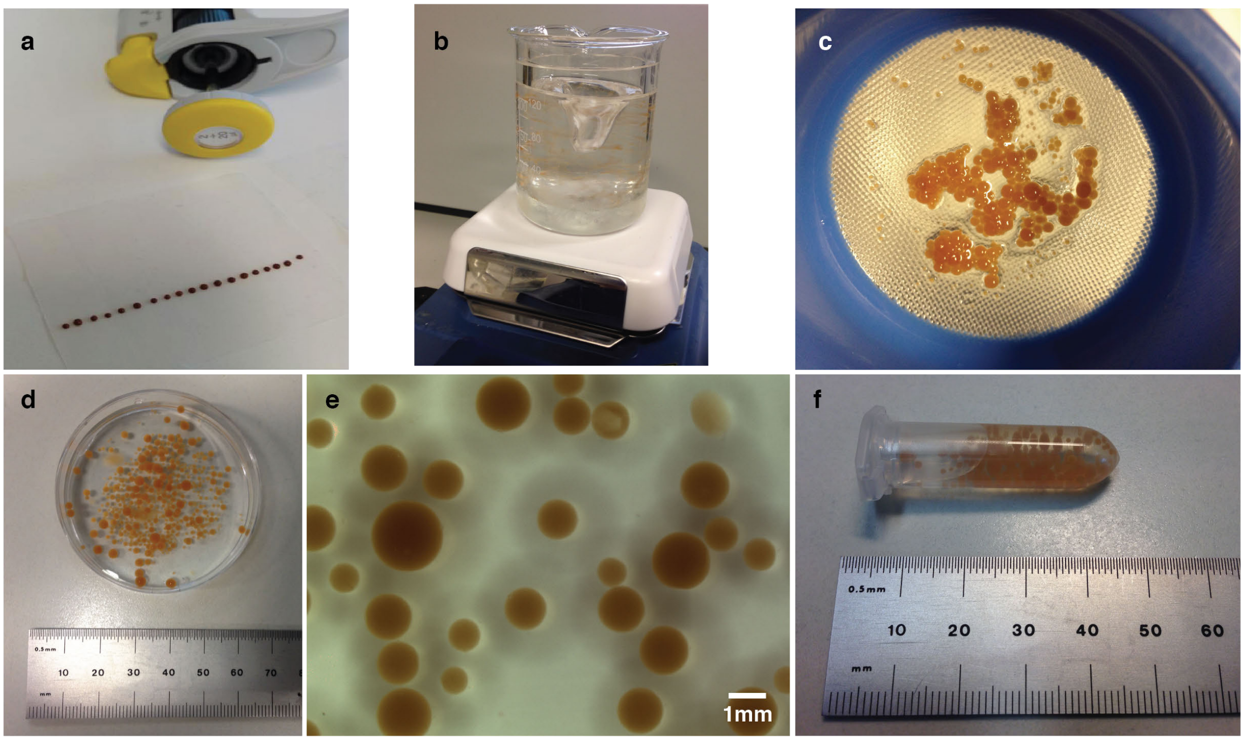

Supplementary Figure 1: Creation of magnetic agarose hemispheres and spheres.

(**a**) Magnetic agarose hemispheres were created by pipetting the hot agarose and magnetic beads mix onto parafilm. (**b**) To create magnetic agarose spheres, the hot agarose and magnetic beads mix were pipetted into stirring mineral oil. (**c**) Magnetic agarose spheres were filtered from the oil by a tea sieve. The magnetic spheres were washed to remove remaining mineral oil. (**d**) Photograph of agarose spheres in water. (**e**) Bright-field image of agarose spheres in water showing range of different spheres diameters. (**f**) Magnetic agarose spheres can be stored in water inside tubes.

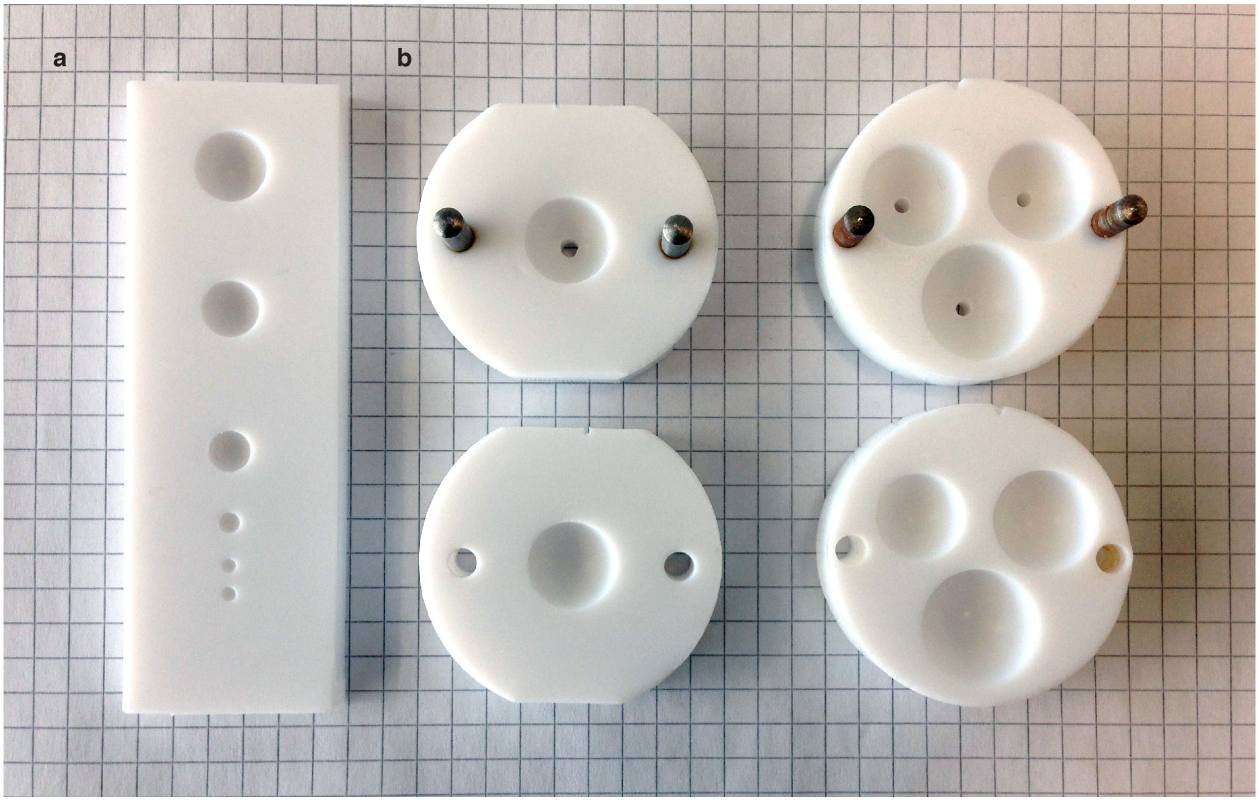

Supplementary Figure 2: Molds to embed samples of various sizes in agarose spheres

(**a**) Photograph of hemispherical molds to embed samples in small agarose spheres for which the surface tension of the liquid agarose is sufficient to form the upper hemisphere. (**b**) Photograph of spherical injection molds to embed samples in agarose spheres that are bigger than 8 mm in diameter (grid size: 5 mm).

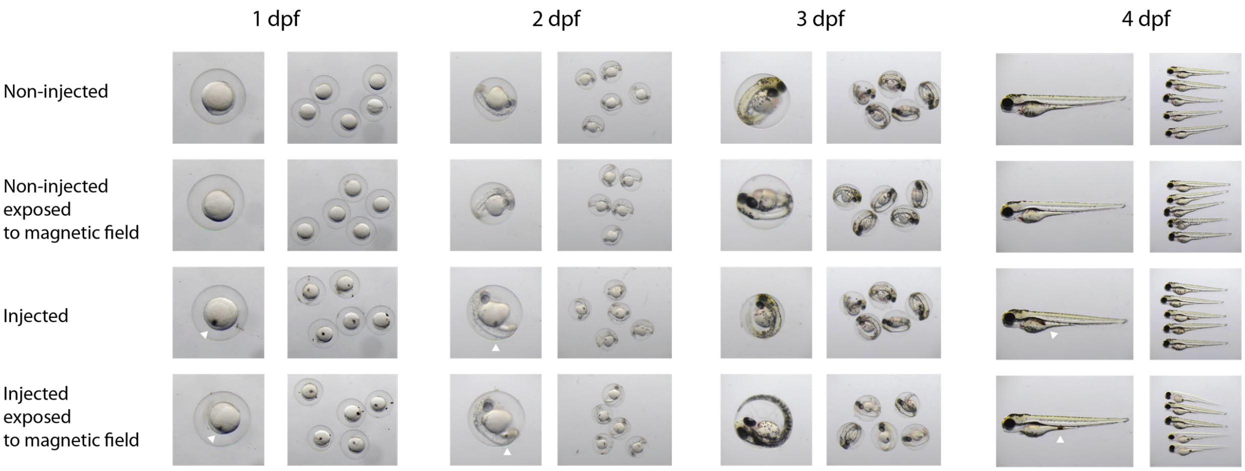

Supplementary Figure 3: Bright-field images of injected and non-injected zebrafish embryos and larvae over four days.

Embryos were injected according to the **Supplementary Methods 1** with 2.8 µm beads (white arrow heads). To investigate whether the injection or the magnetic field has any influence on development, injected and non-injected zebrafish were exposed to magnetic field by placing a permanent magnet close to the dish and monitored over four days. All controls showed no delay in development.

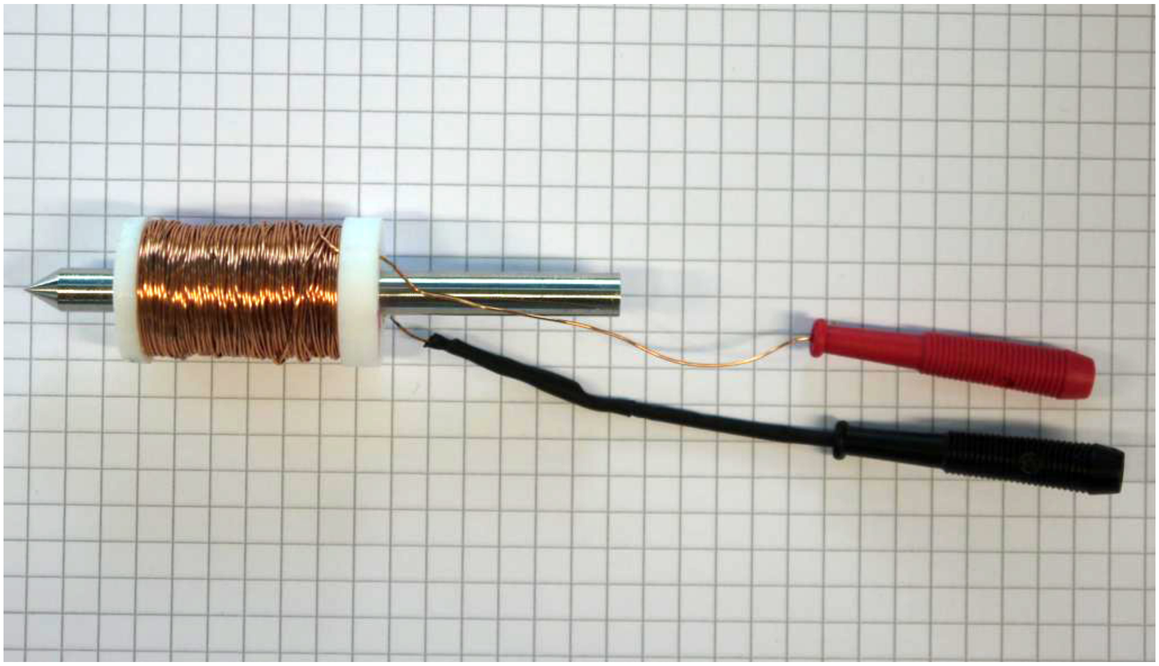

Supplementary Figure 4: Photograph of one of the four custom-built electromagnets.

Shown is the sharpened core inside a Teflon bobbin with the copper coil. Two cables connect to the power supply (grid size: 5 mm).

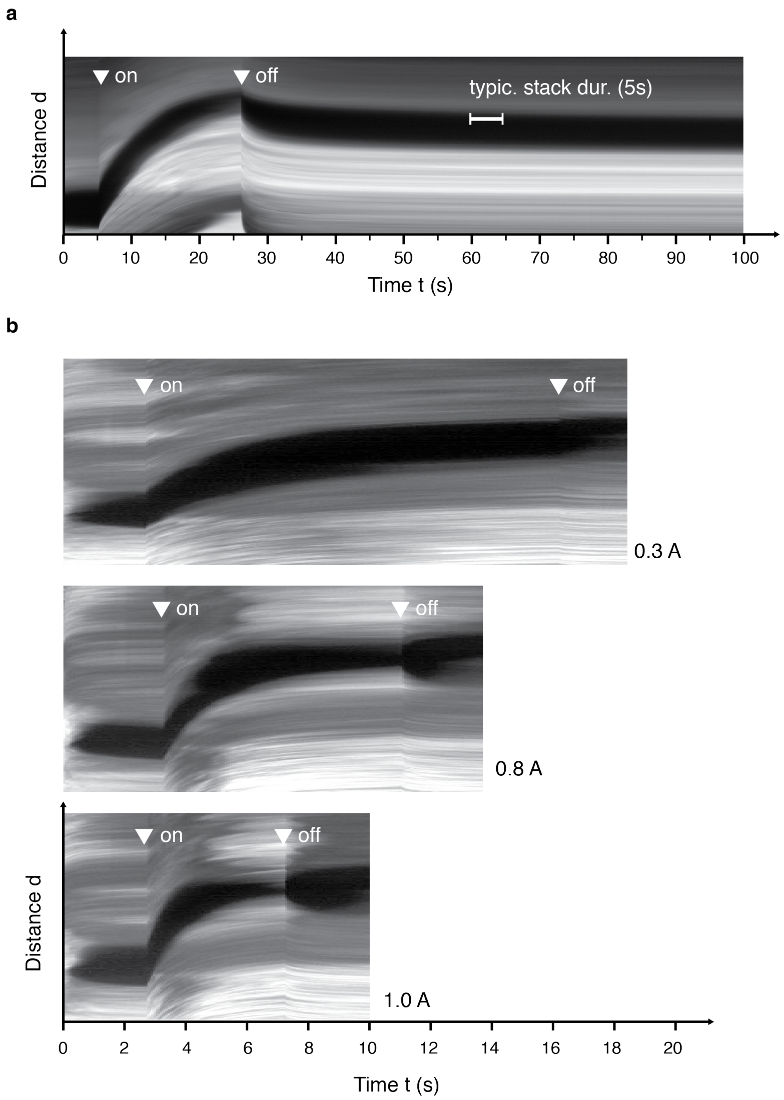

Supplementary Figure 5: Characterization of the rotation with a kymograph

To characterize the rotation of the zebrafish embryos, we generated kymographs of the bright field image sequences showing their reorientation. Since the embryos undergo rotation we generated kymographs by slicing in time along the path of the magnetic beads using FIJI[1]. This path was drawn manually and kept for comparing different currents. (**a**) Kymograph of a reorienting zebrafish embryo. The arrow heads indicate the time-point when the electromagnet was switched on and off again. The rotation of about 109.5° of the zebrafish embryo from one orientation (magnet M1 on) to the next orientation (magnet M3 on) took less than 30 s. After a retraction of the embryo of less than 10 s, the embryo remained stable in its settle position for over a minute, longer than the typical acquisition time for a single image stack in a SPIM setup (a few seconds). (**b**) Rotation of an injected zebrafish with different currents, showing that the rotational speed can be tuned by the applied current. Arrow heads indicate when the electromagnet was switched on and off.

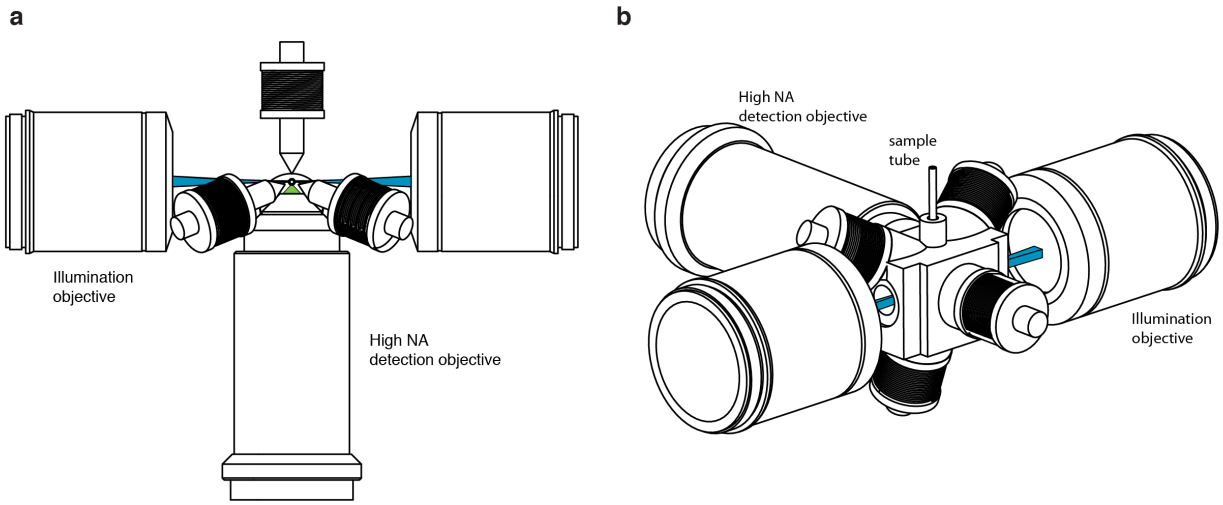

Supplementary Figure 6: Possible configuration for high NA, multi-axes SPIM systems

(**a**) Top view of a possible electromagnets and objective arrangement for high NA, multi-axes SPIM imaging (sample chamber not shown). (**b**) Side view of the same arrangement (sample chamber shown). Lenses shown here: Nikon 16x, 0.8 NA, WD 3 mm for detection and two Olympus 4x, 0.28 NA, WD 29.6 mm for illumination.

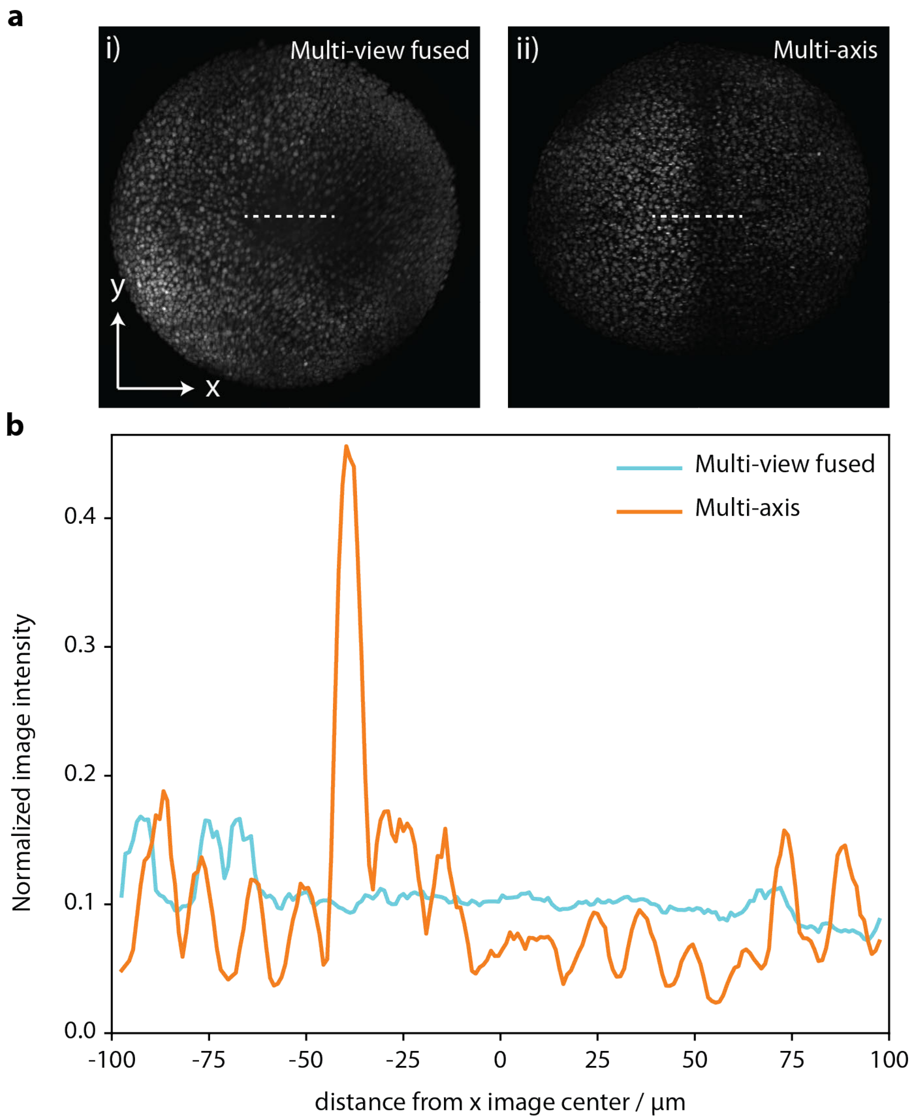

Supplementary Figure 7: Image contrast across the animal pole from line intensity profiles.

(**a**) Maximum intensity projections of i) the multi-view fused dataset and ii) the single-view multi-axis dataset. Estimates of the polar position based on image centroid fail to provide a satisfactory coordinate owing to image attenuation from left to right towards the equator in the single-view multi-axis mode (skewing the pole towards the left of the image). Nevertheless, the embryo was manually centered in the image and so the x,y image center was assumed to lie at the pole in either case. The line along which the line profile is analyzed is shown (200 pixels long - equivalent to 195 µm and approximately a quarter of the embryo equatorial diameter). (**b**) Line profiles for the lines shown in a). The intensity along this line is reported as a normalized intensity (0 to 1) over the entire image.

Supplementary Methods

Supplementary Method 1: Agarose sphere embedding of samples

Step 1: Designing the mold with hemispherical wells
We created a mold with hemispherical wells of different diameters by milling with a ball nose cutter into a piece of Teflon.

Step 2a: Preparing magnetic hemispheres
We put 40 µl of the stock bead solution (10 mg/ml) in an Eppendorf tube. We brought a permanent magnet close to the tube to clump the beads, and we removed the remaining solution using a pipette. The beads were re-suspended in 20 µl of 2% LMA. Using a micropipette, we created 0.5-1 µl droplets of agarose with magnetic beads on a sheet of parafilm. We let it settle for a few minutes and transferred the beads to an Eppendorf tube and stored in water at 4°C.

Step 2b: Preparing magnetic bead spheres
We created the agarose mix as for the preparation of the hemispheres. We pipetted the liquid agarose and magnetic beads mix into stirring mineral oil. The size of the agarose beads could be tuned by the stirring speed and by the size of the pipette tip. To decrease the agarose sphere size, the pipette tip was placed into the stirring oil such that the agarose drop is ripped of the tip by the stirring oil. Depending on the needed size the magnetic agarose spheres were filtered by a tea sieve or a finer sieve. The spheres were washed in the sieve to remove remaining mineral oil and transferred to an Eppendorf tube and stored in water at 4°C.

Step 3: Sample embedding
We pipetted 1.5% low melt agarose into the hemispherical mold of appropriate size such that the hemisphere is filled with one-third volume of the final sphere (choose a well slightly larger than the sample size). We waited until the agarose is partially set. We placed the sample, a mouse embryo for instance, on the agarose along with one magnetic agarose sphere next to it. The magnetic beads should be positioned such that they do not interfere with the imaging. Once placed correctly, we pipetted more agarose on top such that it formed a dome, giving rise to a sphere of desired size. For creating spheres larger than 2 mm, we held the tip of the pipette in contact with the dome for a minute. This ensures uniformity of the sphere. For samples requiring an agarose sphere bigger as 10 mm in diameter we used a spherical injection mold (**Figure 1b and Supplementary Figure 2**) because the surface tension of the agarose was not sufficient to form the upper hemisphere. Once solidified, we removed the sphere from the mold using forceps/spatula. The sphere was examined under the microscope to ensure that both the sample as well as magnetic sphere are properly encased by the surrounding agarose sphere. We kept the agarose spheres with the embedded sample in water/PBS to prevent drying.

Step 4: Imaging
The samples embedded in agarose spheres could be imaged on an upright microscope by placing the sphere in a slightly larger well in the hemispherical mold. They could also be imaged on a SPIM setup by placing the embedded sample in an FEP tube with an agarose plug for support. In both cases, care should be taken that the agarose sphere is submerged in water to reduce friction and can be easily rotated using permanent/electro-magnets.

Supplementary Method 2: Washing and injection of superparamagnetic beads

Step 1: Washing the beads
10µl of the stock bead solution (10 mg/ml) were put in an Eppendorf tube. A permanent magnet close to the tube was used to clump the beads and the remaining solution was removed using a pipette. The beads were re-suspended in 20 µl of distilled water. This process was repeated twice before injecting the diluted bead solution (5 mg/ml) into the embryo.

Step 2: Microinjection
Injections were performed using a micro-injector and injection needles. The opening of the needle was adjusted such that it is not too small for the beads to come out as well as not too large to damage the embryo. The injected beads could in principle cause shadowing artifacts. This problem is negligible since the beads are injected into the opaque yolk, which one should avoid anyway when imaging. The embryos were injected between 2.5 hpf and 4 hpf and the injection needle was inserted from either the vegetal pole or the lateral side. For a sufficient torque, beads were deposited close to the yolk membrane. An extremely low pressure (~10 psi) and long injection duration (~150 ms) were used for the injections in order to avoid dispersion of beads in the yolk. Since the beads sank to the tip of the needle and changed the concentration of the bead solution, the pressure had to be increased for some injections (~45 psi) to prevent blocking of the needle.
Superparamagnetic beads were used, exhibiting magnetic properties only in the presence of a magnetic field [2] since no residual force should be present in the sample after orientation. Beads with a diameter of 2.8 µm were used, which allowed for a rotation of the sample with moderate magnetic fields without translation of the beads within the zebrafish chorion. A volume of 1 nl bead solution corresponding to about 15 ng of beads or about 1000 beads was injected and sufficient to rotate the embryo without damaging the fish.

Step 3: Aggregation of injected beads
After injecting the beads, a strong constant magnetic field was applied with a permanent magnet to attract and clump the beads. This aggregation of beads preserved the single beads from translating through the yolk and eases the rotation.

Step 4a: Embedding zebrafish embryo for SPIM experiments
The zebrafish embryo in E3 buffer was sucked up into a FEP tube with a syringe. To prevent the embryo from being pulled out of the tube the embryo was either embedded in E3 in a tube with an inner tube diameter (1.0 mm), which is a bit smaller than the zebrafish chorion (1.2 mm) or in a bigger tube (inner diameter 1.6 mm) in 1.0% low-melting-point agarose (Sigma). In both cases the embryo (0.8 mm) was still free to move within its chorion.

Step 4b: Embedding zebrafish larvae (5 dpf) for epi-fluorescence microscope experiments
The fish larva with 200 mg/l Tricaine (Sigma) in E3 was sucked into a glass capillary and could be exposed to the magnetic field to rotate it about its anterior-posterior axis.

Supplementary References

1. Schindelin, J. *et al.* Fiji: an open-source platform for biological-image analysis. *Nat. Methods* **9,** 676–682 (2012).

2. Neuman, K. C., Lionnet, T. & Allemand, J.-F. Single-Molecule Micromanipulation Techniques. *Annu. Rev. Mater. Res.* **37,** 33–67 (2007).
